# Video Chat Use and Mealtime Behaviors in Older Adults Before and During the COVID-19 Pandemic

**DOI:** 10.1093/geroni/igab046.2717

**Published:** 2021-12-17

**Authors:** Laura Barre, Tara Young, Sarah Coupal

**Affiliations:** 1 Cornell University, Ithaca, New York, United States; 2 Cornell University, Cornell University, New York, United States

## Abstract

Video chat allows people to connect when not physically together. Using video chat while sharing a meal (VideoDining) may decrease loneliness and improve older adults' nutritional intake. We conducted a cross-sectional online survey study using Amazon Mechanical Turk in June 2020. The objectives were to learn about eating with others, the use of video chat, and interest in VideoDining in older adults during the pandemic. There were 1331 survey attempts with 167 responses meeting the criteria for age (65 years of age or older), U.S. residency, and quality. Participants were 64% male, 77% white, 65% college-educated, and a median age of 67 years (IQR=2 years). Few participants lived alone (17%), yet 76% reported feeling isolated. Eating with others regularly, defined as several times a week or more, declined in the pandemic (44% vs. 59% pre-pandemic, p=0.0002). The use of video chat and eating when video chatting increased during the pandemic versus pre-pandemic (82% vs. 74%, p=0.003; 47% vs. 37%, p=0.0005). The majority of participants said they would VideoDine (50%) or consider trying it (37%). Interest in VideoDining did not vary by age, race, or gender. Participants who used video chat were more likely to say they would VideoDine than participants who had never used video chat (OR=3.1; 95% CI=1.25, 8.35; p=0.02). This data suggests most adults 65 years of age and older, already using the Internet, are experiencing isolation and decreased mealtime commensality during the pandemic. The vast majority are using video chat and are interested in trying VideoDining.

